# Immunological Bases of Paraneoplastic Cerebellar Degeneration and Therapeutic Implications

**DOI:** 10.3389/fimmu.2020.00991

**Published:** 2020-06-02

**Authors:** Lidia Yshii, Chloé Bost, Roland Liblau

**Affiliations:** ^1^INSERM U1043, CNRS UMR 5282, Université Toulouse III, Center for Pathophysiology Toulouse Purpan, Toulouse, France; ^2^Department of Immunology, Purpan University Hospital Toulouse, Toulouse, France

**Keywords:** paraneoplastic cerebellar degeneration, anti-neuronal antibodies, T cell, autoimmunity, immunotherapy, animal model

## Abstract

Paraneoplastic cerebellar degeneration (PCD) is a rare immune-mediated disease that develops mostly in the setting of neoplasia and offers a unique prospect to explore the interplay between tumor immunity and autoimmunity. In PCD, the deleterious adaptive immune response targets self-antigens aberrantly expressed by tumor cells, mostly gynecological cancers, and physiologically expressed by the Purkinje neurons of the cerebellum. Highly specific anti-neuronal antibodies in the serum and cerebrospinal fluid represent key diagnostic biomarkers of PCD. Some anti-neuronal antibodies such as anti-Yo autoantibodies (recognizing the CDR2/CDR2L proteins) are only associated with PCD. Other anti-neuronal antibodies, such as anti-Hu, anti-Ri, and anti-Ma2, are detected in patients with PCD or other types of paraneoplastic neurological manifestations. Importantly, these autoantibodies cannot transfer disease and evidence for a pathogenic role of autoreactive T cells is accumulating. However, the precise mechanisms responsible for disruption of self-tolerance to neuronal self-antigens in the cancer setting and the pathways involved in pathogenesis within the cerebellum remain to be fully deciphered. Although the occurrence of PCD is rare, the risk for such severe complication may increase with wider use of cancer immunotherapy, notably immune checkpoint blockade. Here, we review recent literature pertaining to the pathophysiology of PCD and propose an immune scheme underlying this disabling disease. Additionally, based on observations from patients' samples and on the pre-clinical model we recently developed, we discuss potential therapeutic strategies that could blunt this cerebellum-specific autoimmune disease.

## Introduction

The central nervous system (CNS) can be the target of deleterious cellular and humoral immune responses in context of infectious, degenerative, or autoimmune diseases (1–4). Among these immune-mediated CNS disorders, autoimmune diseases are wide and heterogeneous, occurring both in paraneoplastic and non-paraneoplastic context (1). Paraneoplastic neurological disorders are characterized by acute or subacute neurological manifestations associated with autoantibodies against antigens expressed physiologically by neural cells as well as by tumor cells, so-called “onconeuronal antigens” (5, 6). Although the autoantibodies are considered faithful diagnostic biomarkers of paraneoplastic neurological disorders, their pathogenic contribution, when the target antigens are intracellular, is uncertain (7, 8). In these cases, antigen-specific cytotoxic CD8 T cells that recognize epitopes derived from intracellular proteins in the context of MHC class I presentation are considered the main players causing the neuronal damage (9, 10).

Paraneoplastic cerebellar degeneration (PCD), one of the most common paraneoplastic neurological syndromes (11), represents a heterogeneous group that differs in clinical features, prognosis, associated tumor and associated antibody (7) (Table 1).

**Table 1:** Main autoantibodies reported in paraneoplastic cerebellar degeneration.

**Autoantibodies**	**Target, role, and localization**	**Main associated tumor**	**References**
**PCD with associated autoantibodies**			
Anti-Purkinje cell cytoplasmic antibody-type 1 (PCA1) (anti-Yo)	CDR2 and its paralogue CDR2L, putative neuronal signal transduction proteins, in the cytoplasm of Purkinje cells	Ovarian tumor, breast cancer	(12–14)
ANNA-1 (anti-Hu/HuD)	RNA-binding protein, cytoplasmic, neuronal nuclei	SCLC, other neuroendocrine tumors	(15–17)
Anti-GAD65	Enzyme expressed intracellularly, allowing the conversion of glutamate to GABA in CNS neurons and pancreatic islet cells	Rarely paraneoplastic if not associated with other neuronal autoantibodies: SCLC, neuroendocrine, thymoma, breast cancer, non-Hodgkin lymphoma	(18–22)
Anti-Ca/RhoGTPase-activating protein 26 (anti-ARHGAP26)	Also referred to as oligophrenin-like protein, GTPase-activating protein involved in numerous pathways (in particular in endocytic pathway). In cytosol and at the membrane of Purkinje cells, stellar cells, basket cells, Golgi cells and the granular cells in cerebellum as well as in a subset of neurons in the hippocampus	One case with ovarian carcinoma, one with history of breast cancer and malignant melanoma, one with a B-cell lymphoma, one with prostate cancer, one with gastric adenocarcinoma	(23–27)
Anti-glial fibrillary acidic protein (anti-GFAP)	Major intermediate filament protein of mature astrocytes, localized in the cytoplasm.	Ovarian teratoma, adrenal carcinoma, and others	(28)
Anti-CV2/CRMP5	Cytosolic protein involved in brain ontogenesis by relaying semaphorin 3A signaling. Located predominantly in dendrites of cortical pyramidal neurons, hippocampal CA1 pyramidal cells, Purkinje cells and oligodendrocytes	SCLC, thymoma, gynecological cancer	(29–32)
Anti-metabotropic glutamate receptor 1 (anti-mGluR1)	Main glutamate metabotropic receptor at the cell surface of Purkinje cells; but also widely expressed in the CNS	Hodgkin's lymphoma, prostate adenocarcinoma	(33–36)
Anti-voltage-gated calcium channel (anti-P/Q-type VGCC +/- anti-N-type VGCC)	Membrane high-voltage threshold-activated cation channel, mediating P- and Q-type Ca2+ currents. Important role in glutamatergic neurotransmission. Expressed on Purkinje cells somata and dendrites and abundantly throughout the CNS	SCLC (60%)	(37–39)
Anti-amphiphysin	Thought to regulate exocytosis in synapses and to control the properties of the membrane associated cytoskeleton. It is a cytoplasmic synaptic vesicle-associated protein	When not associated with other neuronal autoantibodies: breast cancer and lung carcinoma	(40, 41)
Anti-dipeptidyl peptidase-like protein 6 (anti-DPPX)	Extracellular regulatory subunit of the Kv4.2 potassium channels at the cell surface of neurons	B cell neoplasm in some patients	(42–45)
Anti-contactin-associated protein 2 (anti-caspr2)	Transmembrane protein. Essential for the clustering of the VGKC subunits Kv1.1 and Kv1.2 at juxtaparanodal regions of myelinated axons and at the axon hillock. Highly expressed in the axons of the granule neurons of the cerebellum	Rarely, thymoma	(46–48)
Anti-γ-aminobutyric acid B receptor antibodies (anti-GABAbR)	Receptor of the main inhibitory neurotransmitter, localized on the neuronal membrane	Around 50% of cases: SCLC, neuroendocrine tumor	(49–51)
AGNA/Anti-SOX1	Developmental transcription factor; preferentially expressed in Bergman glial cell nuclei	Lung cancer	(52, 53)
Anti-Ma2/Ta (PNMA2)	Ma2 could play a role in mRNA biogenesis, localized to structures that resemble nuclear bodies.	Testicular germ-cell tumors.	(54–56)
Anti-Ma1 (PNMA1)	Ma1 could play a role in mRNA biogenesis, localized to structures that resemble nuclear bodies.	Variable: SCLC and non-small cell lung cancer, colon cancer, non-Hodgkin lymphoma, breast cancer	(54, 57)
ANNA-2 (Anti-Ri)	Neuron-specific RNA-binding proteins, widely express in the CNS	Breast and gynecological cancer, SCLC	(58–60)
Anti-Purkinje cell antibody 2 (anti-PCA-2)	Target antigen not known	SCLC (10 cases)	(61)
ANNA-3	Target antigen not known; localized in the neuronal nuclei	SCLC	(62)
Anti-Zic4	C2H2-type zinc finger proteins acting as a transcriptional activator during neurogenesis; in neuronal nuclei.	Hodgkin's lymphoma, SCLC	(63, 64)
Anti-Zic2	C2H2-type zinc finger proteins acting as a transcriptional activator during neurogenesis; in neuronal nuclei.	Mostly associated with anti-Zic4	(65)
Anti-Zic1	C2H2-type zinc finger proteins acting as a transcriptional activator during neurogenesis; in neuronal nuclei.	Always associated with anti-ZIc4	(65)
Anti-Homer protein homolog 3 (anti-Homer-3)	Linking mGluR1 and Homer-3; mainly in the cytosol of Purkinje cells.	One lung cancer but only four cases described	(66, 67)
Anti-Sj/inositol 1,4,5-trisphosphate receptor (anti-Sj/ITPR1)	Mediates intracellular Ca2+ release from the ER calcium storage after activation by mGluR1; mainly located in the membrane of the ER of Purkinje cells.	Five patients described: one breast cancer and no data available for the other four patients	(68, 69)
Anti-carbonic anhydrase-related protein VIII (anti-CARP VIII)	Limit Ca2+ efflux from the ER by reducing the affinity of ITPR1 for inositol 1,4,5-trisphosphate, in Purkinje cells (intracellular).	One melanoma and one ovarian cancer	(70, 71)
Anti-Tr/delta notch-like epidermal growth factor-related receptor (anti-Tr/DNER)	Transmembrane protein involved in the Notch pathway. Highly expressed in Purkinje cell body and dendrites but also in the hippocampus and cortex.	Hodgkin's lymphoma	(72–76)
Anti-tripartite motif-containing protein 46 (anti-TRIM46)	Protein involved in axon specification and outgrowth during early brain development and in the maintenance of microtubules. Specifically localized to the proximal axon.	2 SCLC and one without tumor	(77)
Anti-tripartite motif-containing protein 9 (anti-TRIM9)	Protein expressed widely in the CNS and localizes to cytoplasmic bodies that may be involved in axon guidance.	Lung adenocarcinoma	(78)
Anti-tripartite motif-containing protein 67 (anti-TRIM67)	Expressed in the cytoplasm, exclusively in the cerebellum and retina, involved in neuritogenesis.	Lung adenocarcinoma and melanoma	(78, 79)
Anti- glucose-regulated protein 78 (anti-GRP78)	Plays a role in proliferation, apoptosis and inflammation; expressed on the endothelial cell surface.	SCLC	(80)
Anti-Plasticity-Related Gene 5	Transmembrane protein involved in neurite outgrowth and dendritic spines formation; enriched in hippocampus and cerebellum	Squamous cell lung carcinoma	(81)
Anti-neurochondrin	Leucine-rich neuronal cytoplasmic protein probably involved in signal transduction, in the nervous system	one uterine cancer	(82, 83)
Anti-septin-5	Guanosine triphosphate (GTP)-binding neural protein involved in neurotransmitter exocytosis	non paraneoplastic but only four cases described	(84)
**Example of studies with PCD and without identified autoantibodies**			
	Lung, breast, lymphoma, gastronintestinal, ovary cancer		(85)
	9 patients with PCD and SCLC		(65)
	one thymic carcinoma		(86)
	39 cases with lymphoma, non-SCLC and genitourinary cancers		(87)

Cerebellar degeneration is the dominant presentation but neocortex, limbic system, basal ganglia, spinal cord and the peripheral nervous system can be involved (88). The clinical presentation is partially correlated to the pattern of expression of the target autoantigen in the CNS. Antibodies are mainly directed against intracellular neuronal antigens and, contrary to autoimmune encephalitis associated with antibodies targeting cell surface proteins (such as the NMDA receptor), the pathogenic immune response involves cellular immune mechanisms and irreversible neuronal death. This neuronal death leads to severe and irreversible neurological impairment. Highly specific anti-neuronal autoantibodies in the sera and/or cerebrospinal fluid (CSF) are the key diagnostic biomarkers of PCD. About 50% of PCD cases are related with anti-Yo antibodies, also known as CDR2/CDR2L (cerebellar degeneration-related antigen), making anti-Yo antibodies the predominant autoantibody associated with PCD among the 37 other anti-neural antibodies described (Table 1), such as anti-Hu, anti-Tr, anti-Ri, anti-Ma2, anti-P/Q-type calcium channel, or anti-CV2/CRMP5 (89–91).

The anti-CDR2/CDR2L antibody-associated PCD will be the main focus of the current article. This type of PCD develops mostly in female patients with gynecologic (ovarian and breast) carcinomas that express the Purkinje neuron-specific CDR2 protein (9) and its paralog CDR2L (14), but also in patients with other types of cancers including endometrial, digestive and lung (37, 92–95).

## Expression and Role of CDR2/CDR2L Proteins in Physiological Context

CDR2 and CDR2L are members of the cerebellar degeneration related (CDR) protein family. The Cdr2 gene is widely transcribed and encodes a cytoplasmic leucine zipper protein. The RNA is expressed in almost all tissues but the protein has only been found to be expressed in cerebellar Purkinje neurons, some brainstem neurons, ovarian and mammary tissue, prostate, testis and spermatogonia (96–99). These results suggest that the tissue-specific expression of CDR2 is regulated at a post-transcriptional level.

The biological function of CDR2 remains ill defined, and that of CDR2L even less known. It has been shown that CDR2 inhibits the functions of the oncogene c-Myc, a master regulator of cellular growth and cellular metabolism, through its sequestration in the cytoplasm (100). CDR2 interacts with other proteins involved in signal transduction and gene transcription such as cell cycle-related proteins, and acid-activated serine/threonine protein kinase (100–104). A similar interaction was found with NF-kB, a transcription factor involved in neuronal development and synaptic plasticity (105); and with MRGXm, a transcriptional regulator involved in cell growth and apoptosis (103). These data indicate that CDR2 may be involved in the regulation of cell cycle, at least in part through interactions with c-Myc. Since cells display an impaired proliferation capacity upon CDR2 knockdown, it can be reasoned that CDR2 is required for appropriate mitotic function (101).

Anti-Yo antibodies react against CDR2 (62 kDa protein consisting of 454 amino acids) and CDR2L (a CDR2 paralog consisting of 465 amino acids). CDR2L has a 44.7% sequence identity with CDR2 and contains three potential coiled-coil regions (14). In the cerebellum, both CDR2 and CDR2L are present in the cytoplasm and proximal dendrites of Purkinje cells (96, 100). Recent data, combining co-staining on human cerebellar sections and cultured cancer cells, protein immunoprecipitation and pre-absorption experiments, strongly suggest that under native conditions CDR2L, rather than or in addition to CDR2, is the major target of serum and CSF Yo antibodies (14, 106). However, there is currently no evidence indicating that immunity toward CDR2L actually causes Purkinje cell death.

## Disruption of Immune Tolerance and Initiation of the Autoimmune Process in PCD

### Immune Tolerance and PCD

The expression of CDR2 appears tightly restricted to “immune privileged” sites whereas CDRL2 is transcribed also in the digestive tract. CDR2 has been found to be poorly immunogenic even when expressed in tumors (107, 108). During lymphocytes development in the thymus, tolerance mechanisms delete most autoreactive T cells with high affinity or redirect them to a regulatory phenotype (109, 110). Through “ectopic” expression of a number of tissue-associated self-antigens, the autoimmune regulator (AIRE) acts as a master regulator of central T-cell tolerance by preventing the development of pathogenic autoreactive T cells (111, 112). Interestingly, transcription of both *Cdr2* and *Cdr2l* has been reported in thymic medullary epithelial cells (113). Whether this expression is dependent on AIRE and whether it results in central T-cell tolerance remains unknown at this time. In this context, the observed up-regulation of *AIRE* mRNA expression in ovarian tumors associated with anti-Yo PCD as compared to other ovarian tumors is puzzling (114). Moreover, genes transcriptionally regulated by *AIRE* are enriched among those differentially expressed between anti-Yo-associated *vs*. control ovarian tumors, although the immunological consequences of this differential expression is unknown (114). Collectively, these findings suggest that defects in immune tolerance induction could be implicated in the pathogenesis of PCD.

Central tolerance is only partial since lymphocytes capable of recognizing autoantigens are prevalent, even in healthy individuals (115). Therefore, in order to prevent deleterious autoimmune reactions, peripheral tolerance mechanisms are necessary, among which regulatory FOXP3+ CD4 T cells play a major role. Break of immune tolerance resulting in autoimmunity usually requires a failure of one or several tolerance checkpoints. The autoimmune response against CDR2/CDR2L antigens in the context of PCD is likely multifactorial, involving high CDR2/CDR2L expression in the tumor, a genetic predisposition, and a productive, chronic immune response toward tumor cells, as detailed below.

### CDR2/CDR2L Protein Expression in Tumor and T Cells

The tumor inflammatory microenvironment has been suggested to facilitate the release of intracellular antigens resulting in abnormal exposure of self-antigens to the immune system; this provides an explanation for the numerous autoantibodies produced against intracellular antigens in cancer patients (116). However, PCD is rare even though all ovarian cancer subtypes, regardless of their association with anti-Yo antibodies and PCD, express CDR2/CDR2L (98, 99, 117). Therefore, the mere expression of CDR2/CDR2L by tumor cells is insufficient to trigger autoimmunity against Purkinje cells. The low incidence of PCD may also be linked to the lymphocyte expression of CDR2 (118).

Recently, it has been shown that ovarian tumors associated with PCD and anti-Yo antibodies differ from other ovarian tumors. Indeed, Small and colleagues showed that tumor cells from all 25 PCD patients with anti-Yo antibodies exhibited (likely somatic) mutations and/or gains in *CDR2* and/or *CDR2L* genes, leading to higher protein expression and/or expression of proteins with missense mutations. This high rate of genetic alterations is characteristic of tumors from patients with PCD and anti-Yo antibodies, as they have not been reported in 841 other ovarian carcinomas (119). Moreover, this study demonstrates massive infiltration of PCD tumors with anti-Yo antibodies by activated immune effector cells. This suggests that genetic alterations in tumor cells trigger immune tolerance breakdown and initiation of the autoimmune disease. Comforting this hypothesis, ovarian tumors associated with PCD and anti-Yo antibodies are characterized by a higher and more frequent immune cell infiltration, including CD8 T cells, B cells, plasma cells and mature Lamp+ dendritic cells (DC), known to be associated with more efficient T cell antitumor response (120). The characterization of such DC and whether they contribute to onconeuronal antigen presentation remain to be determined.

### Genetic Basis of PCD

The infrequency of anti-Yo antibody-associated PCD among patients with gynecological cancers could also reflect predisposing factors such as a genetic susceptibility. Hillary and colleagues conducted high resolution HLA class I and class II genotyping in 40 patients with PCD vs. ethnically matched controls (11). They provided evidence for association of the DRB1^*^13:01~DQA1^*^01:03~DQB1^*^06:03 haplotype with ovarian cancer-associated, but not breast cancer-associated, PCD (present in 9 of 29 cases). As HLA class II molecules present antigenic peptides to CD4 T cells, the data suggest that this T cell subset could be a major player in the onset of PCD. Significant findings were also observed with several HLA class I alleles, especially within the HLA-C locus (C^*^03:04, C^*^04:01, and C^*^07:01). These data indirectly suggest the involvement of CD8 T cells or NK cells in PCD pathogenesis. Other genes, in particular immune-related genes, may be at play. A genome-wide association study would be greatly beneficial in identifying these genes which may play varied roles in PCD pathogenesis; albeit, challenging, given the low prevalence of PCD.

## Blood-Brain Barrier Transmigration Into the Cerebellum

Circulating immune cells have to cross the blood-brain barrier (BBB) to get into the CNS, involving distinct trafficking molecules at the surface of the BBB endothelial cells and on immune cells for the sequential transmigration steps: tethering, rolling, capture, adhesion and diapedesis (121). Several surface molecules expressed by T cells, such as P-selectin glycoprotein ligand-1 (PSGL1), activated leucocyte cell adhesion molecule (CD6) and integrins, contribute to these steps. PSGL1 bind to P/E-selectin on endothelial cells and mediates the initial rolling and tethering of CD4 and CD8 T cells (122). Furthermore, the α4β1 integrin interacts with vascular cell adhesion protein 1 (VCAM 1) to form strong adhesion between T cells and the endothelium (123, 124). Under inflammatory conditions, the BBB-endothelial cells up-regulate the expression of adhesion molecules (selectins and cell adhesion molecules of the immunoglobulin superfamily) (121). In experimental autoimmune encephalomyelitis (EAE), a classical animal model of multiple sclerosis, BBB-endothelial cells express CCL2, CCL19, and CCL21, which mediate firm arrest of CCR2+ monocytes and DC as well as CCR7+ CD4 T cells (125). Stimulating chemokine receptors also results in a conformational change of the cell surface integrin molecules providing increased affinity for their ligands (126).

Although cumulative evidence highlights the key role of CD8 T cells in several inflammatory CNS disorders such as PCD, the molecular cues responsible for trafficking of CD8 T cells into the CNS are less known. Interaction between PSGL1 and P-selectin contributes to the recruitment of CD8 T cells from multiple sclerosis patients to brain vessels (127). However, CD8 T-cell transmigration is not affected by blocking interactions between αLβ2/ICAM-1, PECAM-1/PECAM-1, or CCL2/CCR2 (128). Using a murine model of CNS autoimmune neuroinflammation, we showed that the migration of cytotoxic CD8 T cells to the CNS relies on the α4β1-integrin and that VCAM-1 and JAM-B expressed by BBB endothelial cells are likely implicated in this process (129). Targeting this pathway of T-cell trafficking to the CNS may hold promise in neurological diseases other than multiple sclerosis. Indeed, Natalizumab, a humanized mAb against α4 integrin, was tested in a patient with immune checkpoint inhibitor-induced encephalitis resulting in neurological improvement after 2 months of treatment (130) and in a few patients with Susac syndrome (131). It is yet to be determined if the pathogenic immune cells require α4 integrin expression to penetrate into the cerebellum during PCD.

Regional peculiarities may exist for T and B cell migration within the CNS. For example, in 2-day-old piglets the cerebellum was more permeable than the cortical regions to bilirubin (132). Moreover, it has been suggested that the expression of P-glycoprotein, a transporter essential in preventing the BBB penetration of substrates (133), is lower in the BBB of the cerebellum than in the cortex (134). It could therefore be proposed that structural or molecular peculiarities of the cerebellum may contribute to local transmigration of T cells during PCD.

## Immune Mechanisms of PCD

PCD is characterized by the selective and extensive loss of cerebellar Purkinje neurons associated with local inflammatory infiltrates reported in several studies (7, 135, 136). Depopulation of Purkinje cell axons and secondary demyelination is also prevalent. In some patients, immune infiltrates and microglial activation extend beyond the cerebellum. In the cerebellum of PCD patients, the inflammatory infiltrates are composed of CD8 T cells, macrophages, and activated microglia that can form nodules (137–139). CD4 T cells and B cells are either absent or found in small numbers around blood vessels (137). No IgG deposition or complement activation is found in relation to the Purkinje cells (138, 139). In PCD CD8 T cells exhibited an activated phenotype with granzyme B- and perforin-containing cytolytic granules, which are sometimes polarized toward the targeted neurons (140, 141). The fact that Purkinje cells can up-regulate the expression of MHC class I molecules during an inflammatory process may provide the opportunity for CD8 T cells to recognize antigens presented by these neurons (140, 142, 143). We hypothesized that interferon-γ (IFNγ) is likely a part of the local inflammatory milieu as suggested by local up-regulation and nuclear translocation of phosphorylated STAT1 (143). Pre-clinical data have yet to show Purkinje cell destruction by T cells specific for PCD-associated autoantigens. Indeed, Ma1-specific Th1 CD4 T cells can induce encephalomyelitis but failed to induce neuronal degeneration (144). However, in mice, CD8 T cells specific for a model onco-neuronal antigen can kill neurons upon help from antigen-specific CD4 cells (145), with accumulative evidence indicating that cytotoxic T cells are likely final mediators of neuronal injury (146). However, in some instances, complete elimination of Purkinje cells is found in the absence of immune cell infiltration, which is reminiscent of burned out lesions (12, 139). Despite these data strongly arguing that CD8 T cells are the final effector cells involved in Purkinje cell demise, definitive proof is lacking.

A possible direct role of autoantibodies directed against intracellular target has been evoked in early studies (5, 147). However, passive transfer of anti-Yo or anti-Hu antibodies, including through the intracerebro-ventricular route, failed to transfer disease in animal models (5, 148–151). More recently, a direct neurotoxic role of autoantibodies was documented on cerebellar organotypic slice cultures upon incubation with anti-CDR2/anti-CDR2L or anti-Hu antibodies (13, 100, 152–154). Collectively, the studies indicate that, under these experimental conditions, uptake of anti-neuronal antibodies by neurons is possible and may result in neuronal death. From a mechanistic standpoint, both human and rabbit anti-CDR2/CDR2L antibodies applied on cerebellar organotypic slice culture were rapidly internalized by Purkinje cells and led to increased expression levels of voltage-gated calcium channel Cav2.1, protein kinase C gamma and calcium-dependent protease, calpain-2; this resulted in the decrease of arborizations of Purkinje cells (13). It was therefore suggested that this autoantibody internalization causes deregulation of cell calcium homeostasis. This, in turn, leads to neuronal dysfunction, ultimately resulting in destruction of diseased Purkinje neurons (13). Another team highlighted also on rat cerebellar slice cultures that application of anti-Yo positive IgG resulted in marked Purkinje cell death (155). This effect was reversed after adsorption of the anti-Yo antibodies with their 62kDa target antigen (153). As neuronal death preceded mononuclear cell infiltration, the autoantibodies appeared to have a direct pathogenic role. These data raise the question of whether there is anti-Yo antibody penetration across the blood-brain-barrier, or from CSF to tissue, and then inside Purkinje cells in patients with PCD.

## PCD as a Side Effect of Cancer Immunotherapy

As already underlined the occurrence of PCD is infrequent (85), with about 10 cases/year in France (119). The risk for paraneoplastic disease appears to increase with application of immunotherapies for cancer, most notably with use of immune checkpoint blockers (156, 157). Increasing numbers of cases of autoimmune encephalomyelitis developing within days after treatment with anti-PD1 mAb (either as a monotherapy or in combination with anti-CTLA-4) have been recently reported in patients harboring melanoma or other types of cancers (130, 158, 159), possibly identifying paraneoplastic neurological disorders as a side effect of immune checkpoint inhibitors. Moreover, we recently evaluated, experimentally, the possibility to induce PCD after CTLA-4 blockade in a mouse model in which a neo-self-antigen was expressed in both Purkinje cells and implanted breast tumor cells (146). In this context, an enhanced tumor control was obtained at the expense of autoimmune PCD. We showed that the immune checkpoint therapy in this mouse model of PCD elicits T cell migration into the cerebellum and subsequent killing of Purkinje cells (146). Therefore, by blocking an essential inhibitory immunological signal in the mouse model, it is possible to elicit PCD. Interestingly, our recent results indicate that while 84% of anti-CTLA-4-treated mice develop PCD, a much lower proportion of mice developed PCD upon anti-PD1 mAb therapy (unpublished). Recently, it was demonstrated that both anti-PD1 and anti-CTLA-4 antibodies target a subset of tumor-infiltrating T cell populations, resulting in the expansion of exhausted-like CD8 T cells (160). Remarkably, anti-CTLA-4 mAb, but not anti-PD1 mAb, modulated the CD4 effector compartment, specifically inducing the expansion of Th1-like CD4 effector cells (160). These CD4 effector T cells elicited by anti-CTLA-4 mAb improved anti-tumor responses by enhancing CD8 T cell infiltration, and cytolytic CD8 activity, demonstrating that PD-1 and CTLA-4 attenuate T cell activation though distinct molecular and cellular mechanisms.

Building on these observations, it is tempting to hypothesize that patients developing PCD carry polymorphisms, or that tumors harbor alterations, in genes related to the immune regulation pathway. A detailed next-generation sequencing analysis of ovarian or breast tumors associated with PCD-related to anti-Yo antibodies and in circulating T cells could explore whether there are alterations in immune regulation, either locally or systemically, in patients with PCD. In that respect, the transcriptomic profile of 12 ovarian cancers from anti-Yo PCD was compared with public data of 733 control ovarian tumor transcriptomes from The Cancer Genome Atlas database. A total of 5,634 genes were differentially expressed between anti-Yo PCD ovarian tumors and control ovarian tumors; among these genes, two members of the CD28 family—*CTLA4* and *ICOS*—were significantly down-regulated, implying that suppressive functions of T cells could be altered within the anti-Yo PCD ovarian tumor microenvironment (114).

## Overall Immunological Scenario for the Initiation and Development of PCD (Figure 1)

Key questions regarding the site of priming and the antigenic specificity of the pathogenic cerebellum-infiltrating CD8 T cells remain to be answered. Regarding the site of priming, pathological studies indicate the presence of moderate to profound immune infiltrates in the primary tumor or metastases of PCD-associated tumors (12, 119, 135, 140). Importantly, these infiltrates were composed of CD8 T cells with cytotoxic potential, B cells, plasmabasts, and DC-LAMP+ dendritic cells (119). Intriguingly, those tumor infiltrates could sometimes form tertiary lymphoid structures, a feature recently associated with better prognosis and reponse to immune checkpoint blockade (161, 162). The identification of a high expression of CDR2 and CDR2L within anti-Yo PCD-associated tumors as well as a very high rate of *CDR2* and *CDR2L* mutations within the tumor strengthen the hypothesis of local adaptive immune activation against tumor antigens, mutated or not. A similar scenario has been described for patients with paraneoplasic scleroderma, in whom genetic alterations of the *POLR3A* locus and resulting T and B cell responses against the *POLR3A* gene product were detected in 75% of tumors but were absent from control tumors (163). In that regard, the identification of CDR2 antigen-specific CD8 T cells in the blood and CSF of PCD patients favors the hypothesis that these T cells arise as a consequence of anti-tumor immunity (9, 164). However, the TCR repertoire and antigenic specificity of cerebellum-infiltrating CD8 T cells in PCD are still elusive. Leveraging the latest molecular tools to address these questions appears to be the next logical step (4, 165).

**Figure 1:**
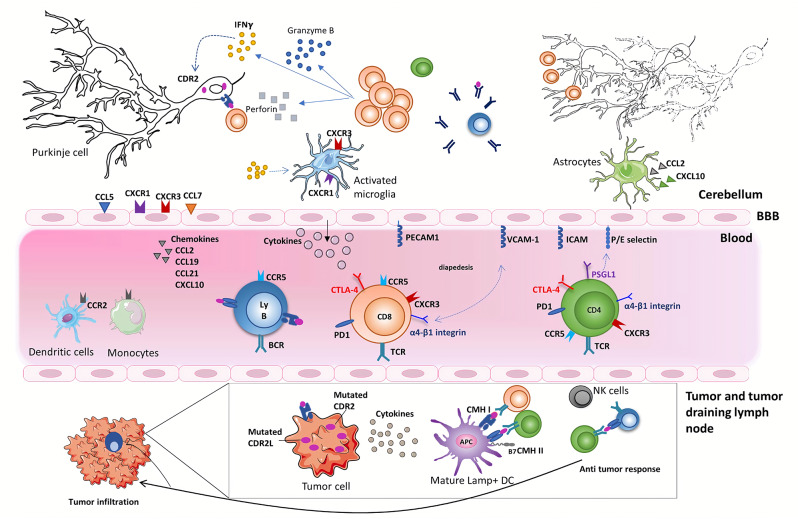
Onconeural antigens such as CDR2 and CDR2L, mutated or not, can be the target of an anti-tumor immune response. Moderate to profound immune infiltrates are found in the primary tumor or metastases of anti-Yo PCD-associated tumors. These infiltrates sometimes form tertiary lymphoid structures. Priming of onconeural antigen-specific CD4 T cells may occur in the tumor-draining lymph nodes or the tumor itself following presentation by local DCs. Differentiation and recruitment of tumor antigen-specific cytotoxic CD8 T-cell likely contribute to the partial tumor control described in patients with PCD. B cells and plasma cells are also found in the tumor. The onconeural antigen-specific T cells reach the CNS after crossing the blood-brain barrier, through a multistep process involving selectins, integrins, and chemokine receptors. The preferential infiltration of the cerebellum in PCD patients could relate to presentation of the onconeural antigens by local APCs and/or to regional peculiarities. In the cerebellum of PCD patients, the inflammatory infiltrates are mostly composed of CD8 T cells, macrophages, and activated microglia. Purkinje neurons are naturally expressing onconeural antigen, such as CDR2 and CDR2L, and up-regulate their expression of MHC class I molecules during an inflammatory process providing the opportunity for CD8 T cells to recognize antigens presented by these neurons. Local CD8 T cells exhibit an activated phenotype with granzyme B- and perforin-containing cytolytic granules. They also secrete locally IFNγ, which has a number of disease-enhancing properties and, thus, is likely a key part of PCD pathogenesis. In particular, IFNγ increases the expression of MHC class I molecules on Purkinje neurons enhances VCAM1 expression on brain endothelial cells and elicits secretion of CXCL10, a ligand of CXCR3 expressed on Th1 cells and activated CD8 T cells. Although antibodies against intracellular onconeural antigens do not appear pathogenic *in vivo*, they could promote antigen presentation to pathogenic T cells.

Several studies in PCD have focused on CD8 T cells, with much less emphasis given to CD4 T cells. In other context CD4 T cells orchestrate functional immune responses by coordinating immune activity (166). CD4 T cells optimize during the priming the cytotoxic response both in quality and scale, by increasing the cytotoxic CD8 T cell motility and migratory capacities (166). The expansion of Th1-like CD4 T cells following blockage of CTLA-4 improves anti-tumor responses by enhancing CD8 infiltration, cytotoxic CD8 T cells activity, and T cell memory formations (160). It is tempting to speculate that in PCD antigen-specific Th1-type CD4 T cells are responsible for initiating the disease, as documented in a rat model (144), and as a consequence, these CD4 T cells are able to recruit and enhance the cytotoxic CD8 T-cell responses.

We have shown in animal models as well as in cerebellar samples from PCD patients that IFNγ signaling occurs locally in Purkinje neurons and surrounding cell types (143). Moreover, CXCL10, an IFNγ-induced chemokine, is present at high level in the CSF of patients with PCD (167). We, therefore, hypothesize that IFNγ stimulates a disease-promoting cascade of events in the cerebellum and could represent a promising therapeutic target in PCD. Indeed, autoantigen-reactive CD8 T cells (and maybe other immune cell types) produce IFNγ locally, which has a number of disease-enhancing properties. IFNγ increases the expression of MHC class I molecules on neurons, including Purkinje cells, which can therefore present self-peptides to the cytotoxic CD8 T cells, promoting their own destruction (143, 168, 169). In addition, IFNγ promotes local immune cell recruitment by enhancing VCAM1 expression on brain endothelial cells and by eliciting secretion of the chemokine CXCL10, a ligand of CXCR3 expressed on Th1 cells and activated CD8 T cells. Importantly, the levels of CXCL10, but not those of CCL2, are elevated in the CSF of PCD patients indirectly suggesting that the CXCL10/CXCR3 axis may contribute to the trafficking of T cells into the cerebellum (167).

The role played by activated microglia and microglia nodules in the disease process is currently unknown. In Rasmussen encephalitis, microglial nodules have been associated with neuronal phagocytosis, following CD8 T cell-mediated brain neuron destruction (170). In human PCD, similar to Rasmussen encephalitis, IFNγ-mediated signaling (STAT1 phosphorylation) occurs in microglial cells, which can further amplify cytokine/chemokine release (143, 170). Further investigation on the role of microglia as an amplifier of inflammation and an executor of neuronal death is needed in PCD.

As already discussed, antibodies against neuronal autoantigens such as anti-Yo antibodies are extremely useful diagnostic biomarkers. Their direct pathogenic potential documented in *in vitro* models is still unproven *in vivo* (5, 13, 149, 153, 171). However, autoreactive B cells could participate in the development of the disease. They can uptake (in an antigen-specific manner due to their surface immunoglobulin), process and present autoantigens to pathogenic T cells. Their scarcity in the cerebellum would, however, rather favor an alternative scenario. For instance, autoantibodies entering the CNS can be up-taken together with their target antigen by resident antigen-presenting phagocytes, a phenomenon that enhances the activation of the incoming effector T cells (172). Therefore, the autoantibodies could cooperate with the T cells and support local autoimmune neuroinflammation.

## Potential Therapeutic Implications

To date, treatment of PCD is empirical and usually relies on 2 pillars: treatment of the underlying cancer and general immunosuppressive drugs. In the absence of large clinical trials, most of the therapeutics conclusions come from observational clinical studies and case reports. Concerning PCD associated with anti-Yo antibodies, corticoids seem ineffective, whereas plasma exchange and rituximab may have provided some benefit (173–176). The efficacy of intravenous immunoglobulins is controversial: suggested in a small proportion of patients for some studies (177, 178) but not confirmed in a larger study (89). Since there is currently no evidence arguing for a direct role of anti-Yo antibodies in Purkinje cell death, we do not address here potential therapeutic strategies aiming at reducing antibodies levels.

Due to the intracellular localization of the CDR2/CDR2L and Hu antigens, as well as the identification of CD8 T cells in close proximity to neuronal cells, cytotoxic T cells are considered to be the final effectors responsible for neuronal loss in PCD (9, 140, 146, 164, 179). These T cells likely contribute to the tumor control outside of the CNS. Therefore, the conundrum is how to selectively target the CNS-targeting immune cells while preserving (as much as possible) the tumor-controlling counterparts. Uncoupling these two simultaneous immune responses is, theoretically, possible provided that the molecular bases of immune cell migration or effector mechanisms at the two sites differ. It is paramount that the treatment of PCD should be started as early as possible since Purkinje cells are post-mitotic cells that do not renew.

The α4β1 integrin is important for both CD4 and CD8 T cell migration to the CNS and a monoclonal antibody targeting the α4 subunit provides important benefits for the treatment of persons with multiple sclerosis. Therefore, one approach could be to initiate anti-α4 integrin therapy early on in the disease process in order to preserve as many Purkinje neurons as possible. One conceptual obstacle is that blood-borne T cells may not be needed to fuel ongoing tissue destruction by tissue-resident T cells. This may explain why, in our mouse model of PCD, blocking α4 integrin early in the disease process did not yield significant benefit (143). Along the same lines, blocking CXCR3 with either monoclonal antibodies or pharmacological compounds is tempting, given the evidence of a role for the CXCL10/CXCR3 axis in PCD. However, to our knowledge, this approach has not yet reached the clinical setting.

We argued earlier that IFNγ secretion by CD8 T cells could sustain a feed forward loop by rendering Purkinje cells vulnerable to direct killing by autoantigen-specific CD8 T cells. If IFNγ plays a non-redundant role in the progression of PCD, this molecule could be targeted therapeutically. Our recent pre-clinical data showing that blocking IFNγ strongly reduces PCD development without eliciting tumor growth rebound are encouraging (143). Moreover, administration of anti-IFNγ antibodies has been tested recently in clinical trials. For instance, Emapalumab, a fully human monoclonal neutralizing anti-IFNγ antibody has been approved for the treatment of primary hemophagocytic lymphohistiocytosis, a syndrome of excessive immune activation and progressive immune-mediated organ damage due to genetic defects in cell-mediated cytotoxicity (180). Taken together, the accumulating evidence from human samples and the mouse model as well as the previous development of approved anti-IFNγ antibody in humans should facilitate the testing of this strategy in patients with PCD.

## Conclusion

The precise mechanisms and pathways involved in the pathogenesis of PCD need to be explored in greater depth. This can be done with the use of bodily fluids (blood and CSF) and tissue from PCD patients and further validated in reductionist mouse models. Key questions remain regarding the antigenic specificity, phenotype, migration of T and B cells infiltrating the tumor and the cerebellar tissue. The molecular dissection of the steps involved in pathogenesis is a pre-requisite for rational development of new therapeutic strategies. In depth investigation of the immune changes in patients suffering from paraneoplastic neurological disorders in the frame of immune checkpoint blockade should provide clues in that respect.

## Author Contributions

LY and CB drafted the work. RL designed the concept and revised the manuscript critically. All authors made substantial contributions to the conception or design of the work and contributed to manuscript revision, read, and approved the submitted version.

## Conflict of Interest

The authors declare that the research was conducted in the absence of any commercial or financial relationships that could be construed as a potential conflict of interest.
